# A small number of point mutations confer formate tolerance in *Shewanella oneidensis*

**DOI:** 10.1128/aem.01968-24

**Published:** 2025-04-10

**Authors:** Megan C. Gruenberg Cross, Elhussiny Aboulnaga, Michaela A. TerAvest

**Affiliations:** 1 Department of Biochemistry and Molecular Biology, Michigan State University 123744 , East Lansing, Michigan, USA; 2 Faculty of Agriculture, Mansoura University 158398, Mansoura, Egypt; University of Nebraska-Lincoln, Lincoln, Nebraska, USA

**Keywords:** formate, microbial electrosynthesis, bicarbonate transporter, SbtA, directed evolution, *Shewanella oneidensis*, *Zymomonas mobilis*

## Abstract

Microbial electrosynthesis (MES) is a sustainable approach to chemical production from CO_2_ and clean electricity. However, limitations in electron transfer efficiency and gaps in understanding of electron transfer pathways in MES systems prevent full realization of this technology. *Shewanella oneidensis* could serve as an MES biocatalyst because it has a well-studied, efficient transmembrane electron transfer pathway. A key first step in MES in this organism could be CO_2_ reduction to formate. However, we report that wild-type *S. oneidensis* does not tolerate high levels of formate. In this work, we created and characterized formate-tolerant strains of *S. oneidensis* for further engineering and future use in MES systems through adaptive laboratory evolution. Two different point mutations in a gene encoding a predicted sodium-dependent bicarbonate transporter and a DUF2721-containing protein separately confer formate tolerance to *S. oneidensis*. The mutations were further evaluated to understand their role in improving formate tolerance. We also show that the wild-type and mutant versions of the putative sodium-dependent bicarbonate transporter improve formate tolerance of *Zymomonas mobilis*, indicating the potential of transferring this formate tolerance phenotype to other organisms.

**IMPORTANCE:**

*Shewanella oneidensis* is a bacterium with a well-studied, efficient extracellular electron transfer pathway. This capability could make this organism a suitable host for microbial electrosynthesis using CO_2_ or formate as feedstocks. However, we report here that formate is toxic to *S. oneidensis*, limiting the potential for its use in these systems. In this work, we evolve several strains of *S. oneidensis* that have improved formate tolerance, and we investigate some mutations that confer this phenotype. The phenotype is confirmed to be attributed to several single point mutations by transferring the wild-type and mutant versions of each gene to the wild-type strain. Finally, the formate tolerance mechanism of one variant is studied using structural modeling and expression in another host. This study, therefore, presents a simple method for conferring formate tolerance to bacterial hosts.

## INTRODUCTION

Microbial electrosynthesis (MES) is a promising technology that utilizes clean electricity and CO_2_ for the green production of biofuels, bioplastics, and platform chemicals (1–3). MES is mediated by electrochemically active organisms, which can utilize reducing power from an electrode and carbon from CO_2_ to generate target molecules through native or engineered metabolic reactions. Three different avenues of MES have been explored to date, each with their own limitations. The first method employs pure cultures of acetogens. At the first glance, acetogens are an attractive MES catalyst because they are autotrophic and some are electrochemically active; however, they suffer from a limited product scope, with acetate being the most abundant product (4–6). Expansion of the product scope is hindered by the small synthetic biology toolbox for acetogens and their slow rate of electron uptake (6, 7). Another limitation of acetogens is the narrow understanding of their extracellular electron transfer (EET) mechanism, which makes it difficult to engineer improvements. At least five mechanisms have been proposed for EET in acetogens, but experimental evidence supporting any of these mechanisms is lacking, and it is not known whether a single EET mechanism is shared by all electrochemically active acetogens (8).

Some of these issues are addressed by the second major approach to MES, replacing the acetogen biocatalyst with mixed cultures. Mixed cultures have more success in producing a wider variety of products, although acetate production remains high (9–11). However, utilizing mixed cultures for MES is plagued by off-target methane production due to rising methanogen populations in the reactors over time (5). A third approach uses model organisms in conjunction with exogenous electron carriers for MES. Model organisms like *E. coli* are more versatile because of their well-understood metabolism and abundance of synthetic biology techniques (12). However, scaling up MES reactions that require exogenous electron carriers is likely not feasible due to their cost (13, 14). To make MES commercially competitive, a better microbial catalyst with well-understood EET mechanisms, metabolism, and synthetic biology tools is needed. One promising candidate is the electroactive bacterium *Shewanella oneidensis*. The discovery of the inward electron transfer pathway in this organism suggests that if coupled to a C1 assimilation pathway, *S. oneidensis* would likely serve as an improved MES biocatalyst (15–17).

*S. oneidensis* is a heterotrophic facultative anaerobe with the ability to utilize a wide variety of terminal electron acceptors for respiration, including electrodes (18, 19). Extracellular electron transfer is made possible by the Mtr pathway, which includes the Mtr complex (MtrCAB), CctA, FccA, and CymA (20). Quinone-linked oxidoreductases such as NADH dehydrogenase, formate dehydrogenase, and lactate dehydrogenase pass electrons obtained from their respective oxidation reactions to inner membrane quinones (21–23). CymA, an inner membrane tetraheme cytochrome *c*, receives electrons from the quinone pool and subsequently passes them to periplasmic *c*-type cytochromes FccA and CctA to traverse the periplasm (24–26). On the other side of the periplasm, the cytochromes transfer the electrons to the outer membrane Mtr complex (MtrCAB), which transports the electrons to the outer surface of the cell (20). The Mtr complex is composed of a porin (MtrB) that spans the outer membrane and encases two cytochrome *c* units, one that interfaces with the periplasm (MtrA) and one that interfaces with the extracellular space (MtrC) (27, 28). Once outside the cell, electrons can either directly reduce extracellular electron acceptors or be shuttled via extracellular flavins (18, 19).

Its well-studied, reversible Mtr pathway makes *S. oneidensis* a promising host for MES because it can efficiently exchange electrons with an electrode; however, this organism cannot fix CO_2_. *S. oneidensis* requires engineered CO_2_ fixation for its full potential as an effective MES biocatalyst to be realized. There are multiple potential methods for CO_2_ fixation, with two possibilities for initial conversions: carboxylation, such as in the Calvin cycle and the reductive tricarboxylic acid cycle, and reduction, such as in the Wood–Ljungdahl pathway and the reductive glycine pathway (29–31). Pathways that first reduce CO_2_ to formate support higher biomass yields due to more effective utilization of ATP (29, 31). Because of this, engineering CO_2_-fixation in *S. oneidensis* via a reduction-first pathway would be a promising strategy for generating an MES-compatible host. CO_2_ can be reduced to formate via reversible formate dehydrogenases (32, 33), and formate can be further assimilated through different pathways such as the reductive glycine (rGly) pathway, the serine cycle, and reversed activity of pyruvate-formate lyase (34). Additionally, formate-assimilating *S. oneidensis* could also be utilized for MES when an electrode with adsorbed formate dehydrogenase is supplied because such electrodes freely interconvert CO_2_ and formate with low overpotentials (33, 35). In this work, we report that *S. oneidensis* is not naturally tolerant of high formate concentrations. This observation suggests that greater formate tolerance is required if a CO_2_-reducing electrode is used or a reduction-first CO_2_ fixation pathway is expressed. Therefore, we utilized adaptive laboratory evolution to create formate-tolerant strains of *S. oneidensis* as a first step toward developing an MES-compatible host. This adaptation was conducted aerobically due to our interest in implementing the rGly pathway for formate assimilation. The rGly pathway requires 2 ATP molecules to assimilate one formate to one pyruvate; however, under anaerobic conditions, only one ATP is generated from pyruvate via substrate-level phosphorylation. Aerobic cultivation allows for operation of the tricarboxylic acid cycle that can supply sufficient ATP for the operation of the rGly pathway via oxidative phosphorylation. We found two genes that are responsible for increasing formate tolerance in this organism and explore their application toward a future CO_2_-fixing strain of *S. oneidensis*. We also show that transfer of one formate tolerance mechanism to another host, *Zymomonas mobilis*, is possible, demonstrating that conferral of formate tolerance to other species is possible through the expression of one gene.

## MATERIALS AND METHODS

### Strains and plasmids

Strains and plasmids used in this study are listed in Table S1. Plasmids for mutant gene overexpression were prepared by cloning the WT or mutant gene from WT or mutant genomic DNA via PCR with primers (Table S2) that added regions homologous to the backbone vector pRL814 (36) at the NdeI and HindIII restriction sites. WT copies of SO_3758 and SO_1320 were amplified from WT *S. oneidensis*, SO_3758_A269T was amplified from MGC002, SO_1320_V106I was amplified from MGC007, SO_1320_V106F was amplified from MGC003, and SO_1320_S52I was amplified from MGC008. Amplified genes were purified via the Wizard PCR clean-up kit (Promega). The pRL814 (containing green fluorescent protein (GFP) under control of *P*_T7A1-O34_) backbone was digested with NdeI and HindIII-HF (NEB). The digested backbone and amplified genes were ligated using NEBuilder HiFi DNA Assembly Master Mix (NEB) and transformed into chemically competent *E. coli* WM3064, a 2,6-diaminopimelic acid (DAP) auxotroph. Plasmids were transferred to WT *S. oneidensis* MR-1 via conjugal transfer using *E. coli* donor WM3064 (27) or into *Z. mobilis* ZMDR (37) using *E. coli* donor WM6026 as described previously (38, 39).

### Culturing

*S. oneidensis* strains were pre-cultured in 2 mL LB with the appropriate antibiotic overnight at 30°C with shaking at 250 rpm. The pre-cultures were washed three times and resuspended in basal M5 -caa medium (1.29 mM K_2_HPO_4_, 1.65 mM KH_2_PO_4_, 7.87 mM NaCl, 1.70 mM NH_4_SO_4_, 475 µM MgSO_4_·7H_2_O, 10 mM HEPES, and pH 7.2). Minimal lactate medium was used for all experimental *S. oneidensis* cultures, unless otherwise stated, and comprised basal M5 -caa, supplemented with Wolfe’s mineral solution, Wolfe’s vitamin solution without riboflavin, and 20 mM D,L-lactate. Formate and isopropyl β-D-1-thiogalactopyranoside (IPTG) were added to the appropriate cultures at the specified concentrations. Washed pre-cultures were used to inoculate the medium to an initial OD_600_ of 0.02. Cultivation took place at 30°C with shaking at 250–275 rpm. Culture volumes are specified for each experiment.

Anaerobic cultures used high HEPES minimal lactate media, which is the same as minimal lactate media, except the HEPES concentration is increased to 100 mM. Anaerobic cultures were carried out in serum bottles with a total volume of 80 mL high HEPES minimal media supplemented with 50 mM fumarate as the electron acceptor. An anaerobic atmosphere was achieved by sparging the bottles with nitrogen through the media for 10 minutes following sterilization. Bottles were inoculated to an OD_600_ of 0.2 with washed precultures, as described above.

*Z. mobilis* strains were pre-cultured overnight in the ZRMG liquid medium (1% yeast extract, 2% glucose, 15 mM KH_2_PO_4,_ and 100 µg/mL spectinomycin) at 30°C, with shaking at 250 rpm. Formate tolerance growth experiments were conducted using the ZRMG medium and were inoculated to an initial OD_600_ of 0.01 from the pre-culture. Formate was added at the specified concentrations. Growth of *Z. mobilis* strains was conducted with basal expression of GFP, SO_3758, or SO_3758_A269T from pRL814. Cultivation was performed in 200 µL of medium at 30°C, with shaking at 250–275 rpm. Growth of *S. oneidensis* and *Z. mobilis* strains in 200 µL cultures was monitored on a H1M BioTek Plate Reader.

### Evolution of formate tolerance and genome sequencing

Formate tolerance in *S. oneidnesis* was evolved by continuously subculturing the parent strains (WT, JG2957, JG2955) in 2 mL minimal lactate medium with increasing concentrations of formate. Pre-cultures of the parent strains were prepared as described above and were used to inoculate three 2 mL cultures of minimal lactate medium with 1 mM formate to an initial OD_600_ of 0.02. The three or four cultures for each parent strain represented a separate evolution line: line A, line B, line C, and line D. Cultures were incubated at 30°C with shaking at 250 rpm. Aliquots of 2 µL were taken daily to monitor OD_600_ using an Eppendorf G1.0 microcuvette and an Eppendorf BioSpectrometer. When cultures reached an OD_600_ of 0.2 or higher, they were subcultured into a fresh 2 mL culture with minimal lactate medium and 5 mM formate. Strains were continuously cultured in this way, with the formate concentration increasing in each subculture. The formate concentration progression was as follows: 1 mM, 5 mM, 10 mM, 20 mM, 30 mM, 40 mM, 50 mM, 60 mM, 70 mM, 80 mM, 90 mM, and 100 mM. Strains spent only one round of culturing in each formate concentration before moving to the next highest concentration. Cryogenic stocks were prepared from each of the populations when the 100 mM cultures reached an OD_600_ of 0.2 or higher. Cryogenic stocks from each evolution line were streaked onto LB agar plates, and multiple single colonies from each evolution line were saved as pure cryogenic stocks. Genome extraction, library preparation, and Oxford Nanopore sequencing were performed by using Plasmidsaurus. Sequencing data are available upon request.

### Bioinformatics

Raw genome sequencing reads were trimmed using Porechop v0.2.4 (https://github.com/rrwick/Porechop.git) to remove Nanopore sequencing adapters. Trimmed sequencing reads were aligned to the *S. oneidensis* MR-1 reference genome (accession: NC_004347.2) and reference megaplasmid (accession: NC_004349.1) and analyzed for mutations using Breseq v0.38.0 (40). Deviations of the evolved strains from the reference sequences were compared to the deviations of the parent strains from the reference sequences. Deviations that appeared in the evolved strains but not in the parent strains are compiled in Table S3 and classified as mutations. Mutations that appeared in multiple evolved strains were of particular interest. A sodium-dependent bicarbonate transporter (SO_3758) and a DUF2721-containing protein (SO_1320) were experimentally investigated for utility in formate tolerance.

Amino acid sequences of WT and variant versions of SO_3758 were aligned to the sodium-dependent bicarbonate transporter SbtA from cyanobacteria *Synechocystis* sp. PCC 6803 substr. Kazusa (accession: P73953) using T-coffee simple multiple sequence alignment (41). Accession numbers for SO_3758-like proteins used for multiple sequence alignment are shown in Table S4. Accession numbers for SO_1320-like proteins used for multiple sequence alignment are shown in Table S5.

Tools DeepGO and DeepTMHMM were used to analyze the amino acid sequence of SO_1320 (42, 43). AlphaFold v2.3.2 was used to predict structures of SO_3758_A269T, SO_1320_V106I, SO_1320_V106F, and SO_1320_S52I (44). Predicted structures for SO_3758 (accession: Q8EAY3) and SO_1320 (accession: Q8EHA9) were downloaded from the AlphaFold database (https://alphafold.com/). Ligand-binding prediction was performed with AutoDock Vina v1.1.2 (45). PyMOL v2.5.8 was used for visualization of protein and ligand models, identifying polar interactions between the ligand and surrounding amino acids, and calculating distances between interacting atoms (46).

## RESULTS

Two *S*. *oneidensis* strains were used in the testing and evolution of formate tolerance: wild-type (WT) and JG2957 (23). *S. oneidensis* strain JG2957 has the genes encoding three major FDHs deleted (*fdhA1B1C1*, *fdhA2B2C2*, and *fdnGHI*) and was included to prevent oxidation of formate to CO_2_ and provide a deeper understanding of formate tolerance.

**Fig 1:**
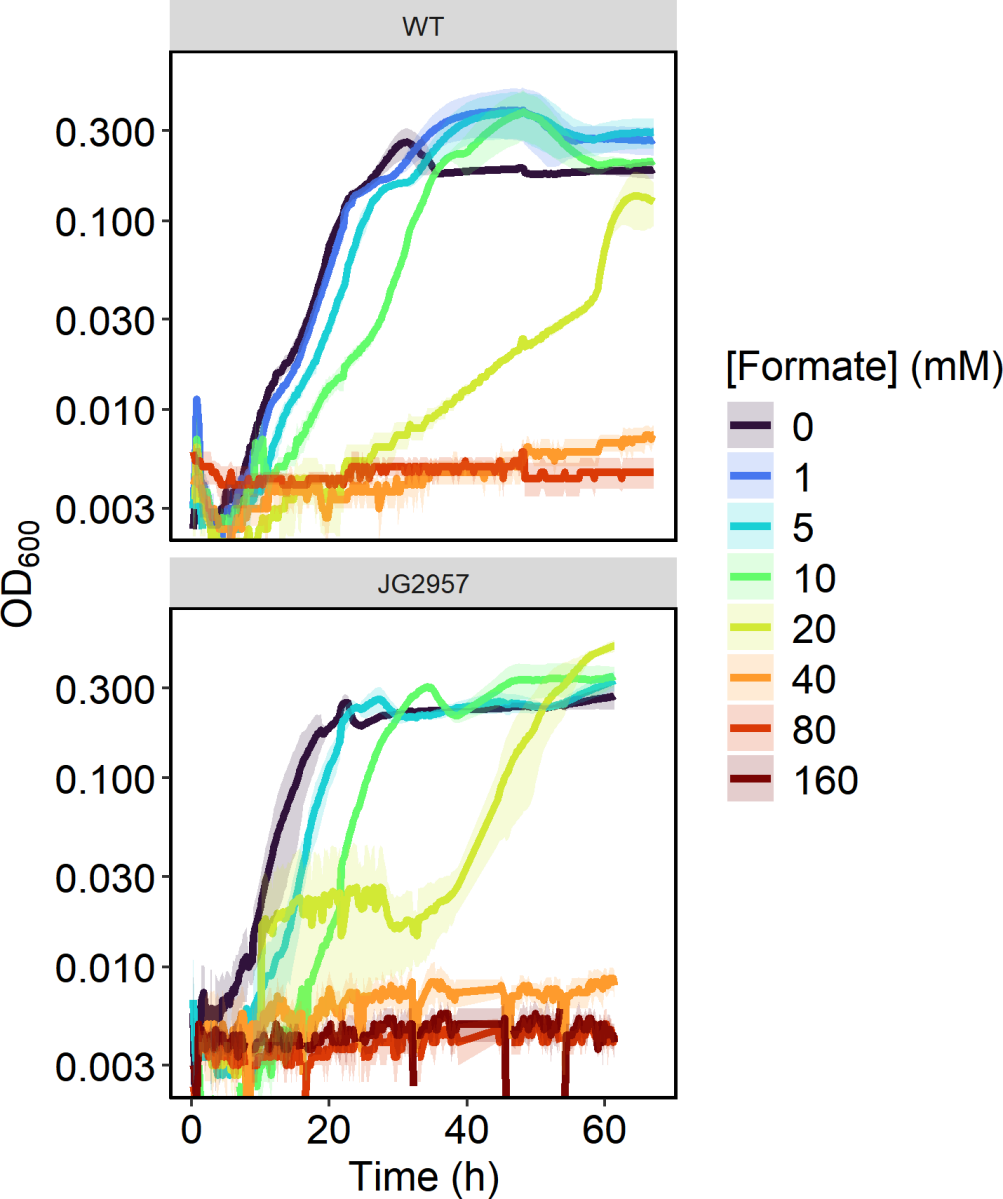
Aerobic growth curves of MR-1 and JG29597 in 200 µL minimal medium with 20 mM lactate and varying concentrations of formate. All conditions were tested in triplicate, and standard deviation values from replicates are displayed with transparent ribbons.

First, to assess the native formate tolerance of *S. oneidensis*, WT and JG2957 were cultivated in 200 µL minimal lactate medium with varying concentrations of formate (Fig. 1). Both strains had the shortest lag phase in media without formate. With even as little as 1 mM formate, growth was impacted. With increasing formate concentrations, the strains’ lag phase also increased. Both strains were only capable of tolerating up to 10 mM formate. The low formate tolerance in these strains could present an issue in an *S. oneidensis* strain with CO_2_ reductase activity. We confirmed that the inhibitory effect of formate was not due to the decreased buffering capacity of the media. We found that minimal lactate medium supplemented with 100 mM formate had a pH of ~7.0. Tolerance of *S. oneidensis* MR-1 to formate was similarly low under anaerobic conditions (Fig. S1).

**Fig 2:**
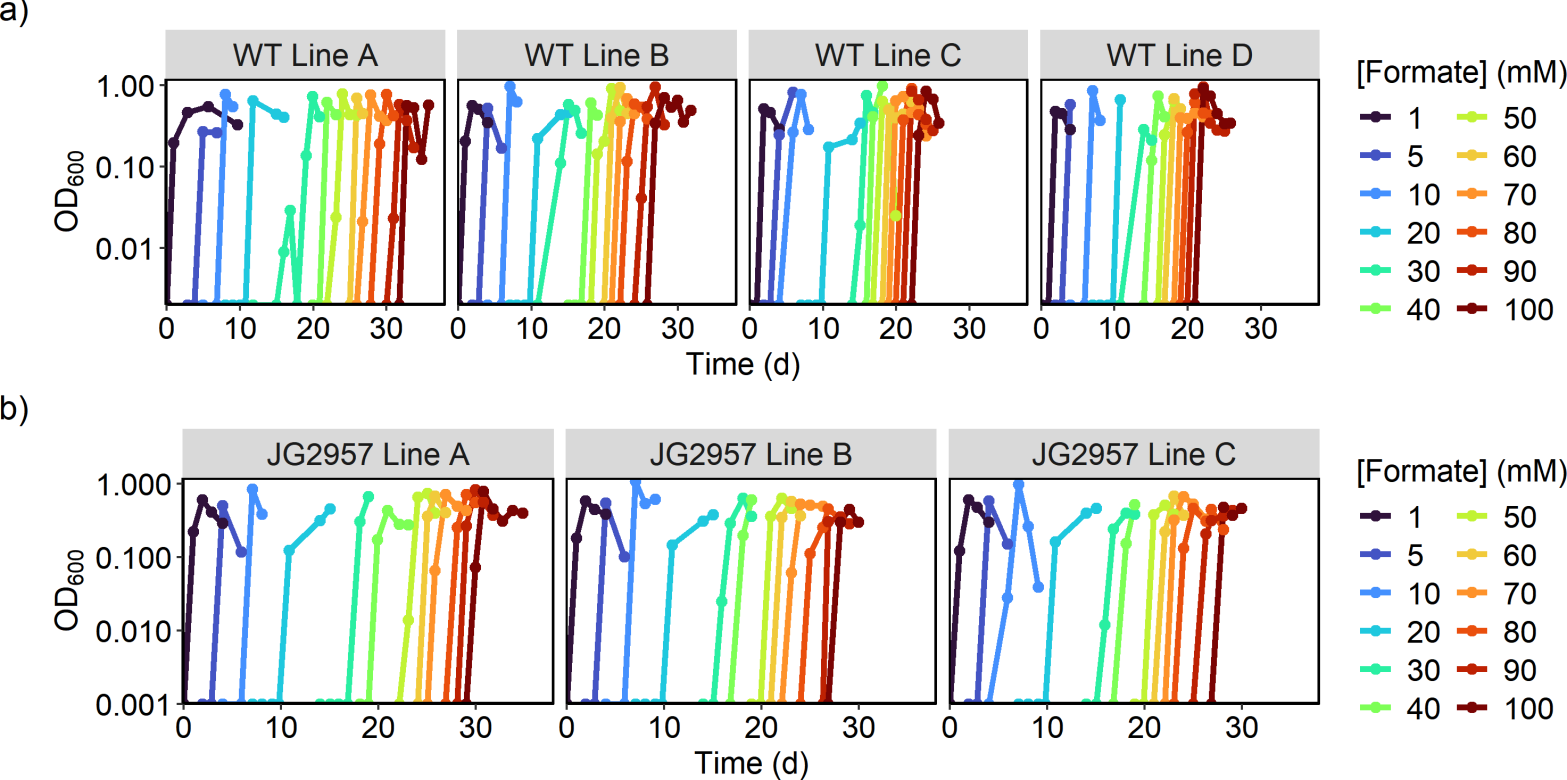
Growth curves of directed evolution subculturing in increasing formate concentrations of parent strains (a) WT and (b) JG2957. Each panel displays growth curves of a replicate evolution line. When the formate concentration reached 100 mM, evolved populations were saved for further analysis.

Formate toxicity can be a result of cytochrome *c* oxidase inhibition and/or reduction in proton motive force due to cytoplasm acidification from the protonated acid (47–49). Because these issues are not easily solved with a genetic engineering approach, we opted to generate a formate-tolerant strain of *S. oneidensis* through adaptive laboratory evolution. This was accomplished through continuous subculturing of the parent strains (WT and JG2957) in minimal lactate medium, while increasing the formate concentration in the medium for each subculture, to improve tolerance to 100 mM formate. Multiple separate evolutions were conducted for each parent strain. Surprisingly, tolerance to 100 mM formate evolved within 30 days in seven evolution lines (Fig. S2), with the subculturing frequency increasing in the later stages of the evolution, indicating acquired mutation(s) assisting with formate tolerance (Fig. 2).

**Fig 3:**
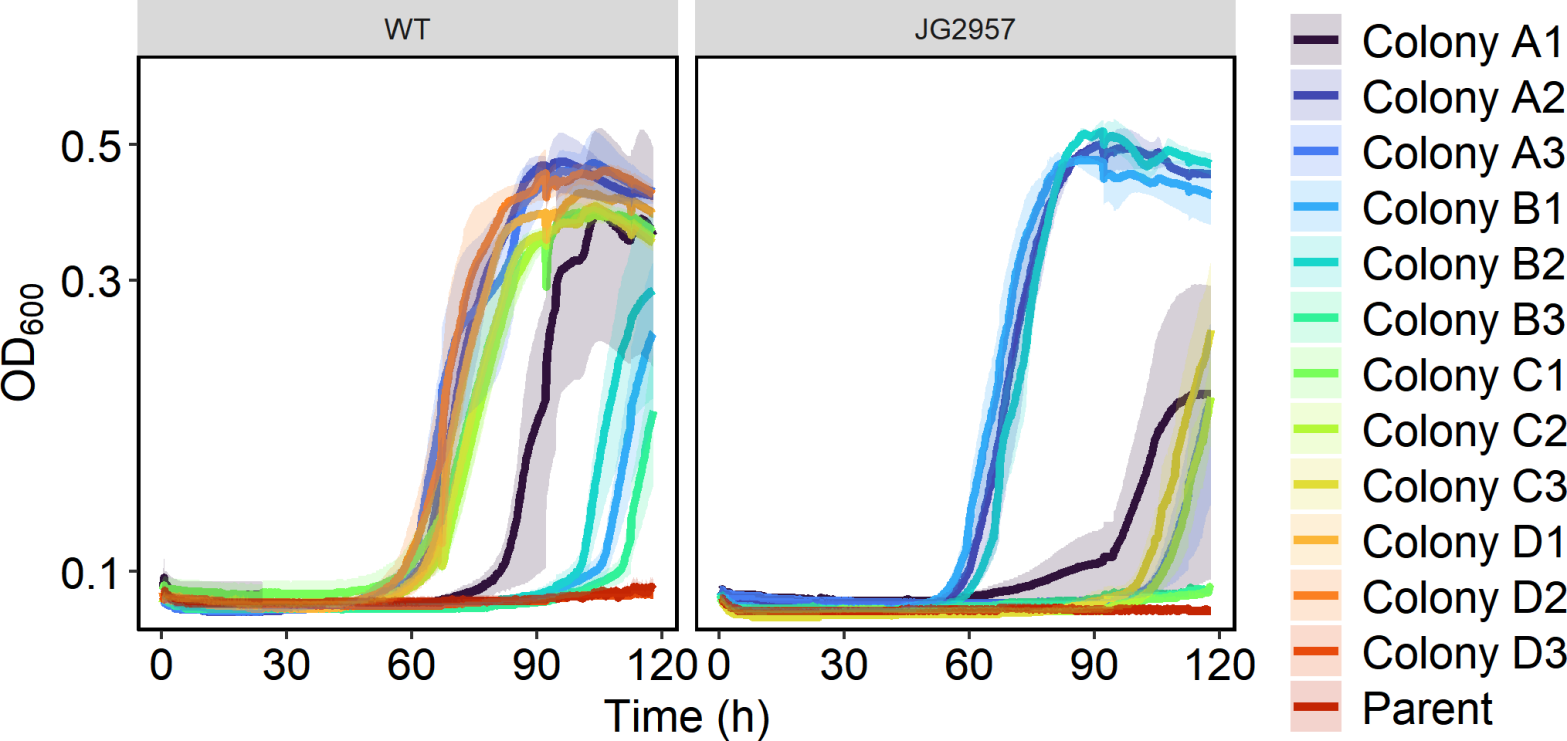
Growth curves of single colonies in triplicate isolated from evolution lines at 100 mM formate compared to their associated parent strain. Strains were cultured in 200 µL of minimal medium with 20 mM lactate and 100 mM formate. Each condition was tested in triplicate, and the standard deviations are displayed as transparent ribbons.

Single colonies from the seven evolution lines capable of growth on 100 mM formate were isolated. Their formate tolerance was compared to that of their associated parent strain by culturing in 200 µL minimal lactate medium with 100 mM formate in triplicate (Fig. 3). Both parent strains were incapable of growth on lactate when supplemented with 100 mM formate, while almost all the isolated evolved colonies were capable of growth, although not without a long lag phase of 60–90 hours. The evolutions from WT resulted in four lines (A, B, C, and D) that showed different growth phenotypes, with lines A, C, and D having a shorter lag phase than line B. Less clear patterns emerge from strains evolved from JG2957. Two of the line A colonies and one line B colony could grow well under the conditions tested, while the other colonies from those lines more closely resembled the growth patterns of colonies from line C.

**Fig 4:**
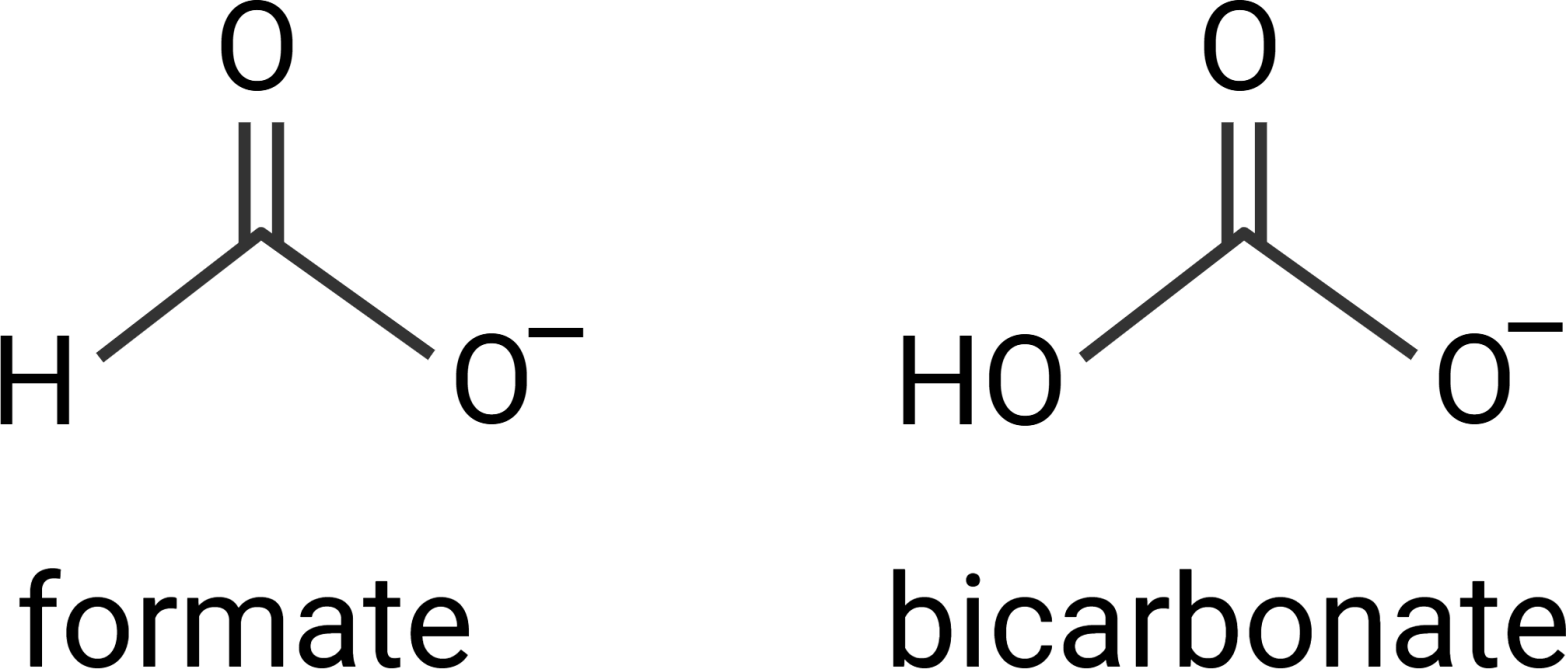
Chemical structures of formate and bicarbonate.

One colony from each line was selected for further analysis. These seven new formate-tolerant *S. oneidensis* strains were renamed MGC001 through MGC007 (Table S1). To determine the mutations that enabled increased formate tolerance, the genomes of the seven formate-tolerant strains and the two parent strains were sequenced. Raw sequencing reads were processed with porechop to remove sequencing adapters and breseq to align the reads to the reference sequences and identify mutations. Mutations that did not exist in the parent strain were flagged (Table S3). A transition mutation of G to A in SO_3758 (new locus tag: SO_RS17510), encoding the core domain of a putative Sbt-like sodium-dependent bicarbonate transporter, occurred in three of the seven formate-tolerant strains (MGC002, MGC005, and MGC006). This resulted in the amino acid sequence change A269T, replacing a nonpolar, hydrophobic residue with a bulkier, polar, hydrophilic residue. Considering that formate and bicarbonate have very similar structures (Fig. 4), we hypothesized two potential effects of this variation. First, the amino acid change could have provided greater specificity toward formate and provided SO_3758 with formate efflux activity, preventing toxic intracellular accumulation. Second, the change may have decreased formate specificity and prevented SO_3758 from importing toxic levels of formate.

In three of the remaining formate-tolerant strains (MGC003, MGC004, and MGC007), a mutation in SO_1320 (new locus tag: SO_RS06120), encoding a protein of unknown function, was observed. MGC007 had a G-to-A transition mutation, resulting in a V106I amino acid sequence change, and MGC003 and MGC004 had a G-to-T transversion mutation, resulting in a V106F amino acid sequence change. We also carried out preliminary adaptive laboratory evolution using *S. oneidensis* parent JG2955 (23), which has genes encoding two of the three formate dehydrogenases deleted (*fdhA1B1C1* and *fdhA2B2C2*). Interestingly, one strain (MGC008) isolated from this evolution also had a mutation in SO_1320, but in residue 52, where a serine was converted to an isoleucine. The two residue 106 variations resulted in a bulkier amino acid in that position, and the S52I change replaced a polar, hydrophilic residue with a nonpolar, hydrophobic residue.

**Fig 5:**
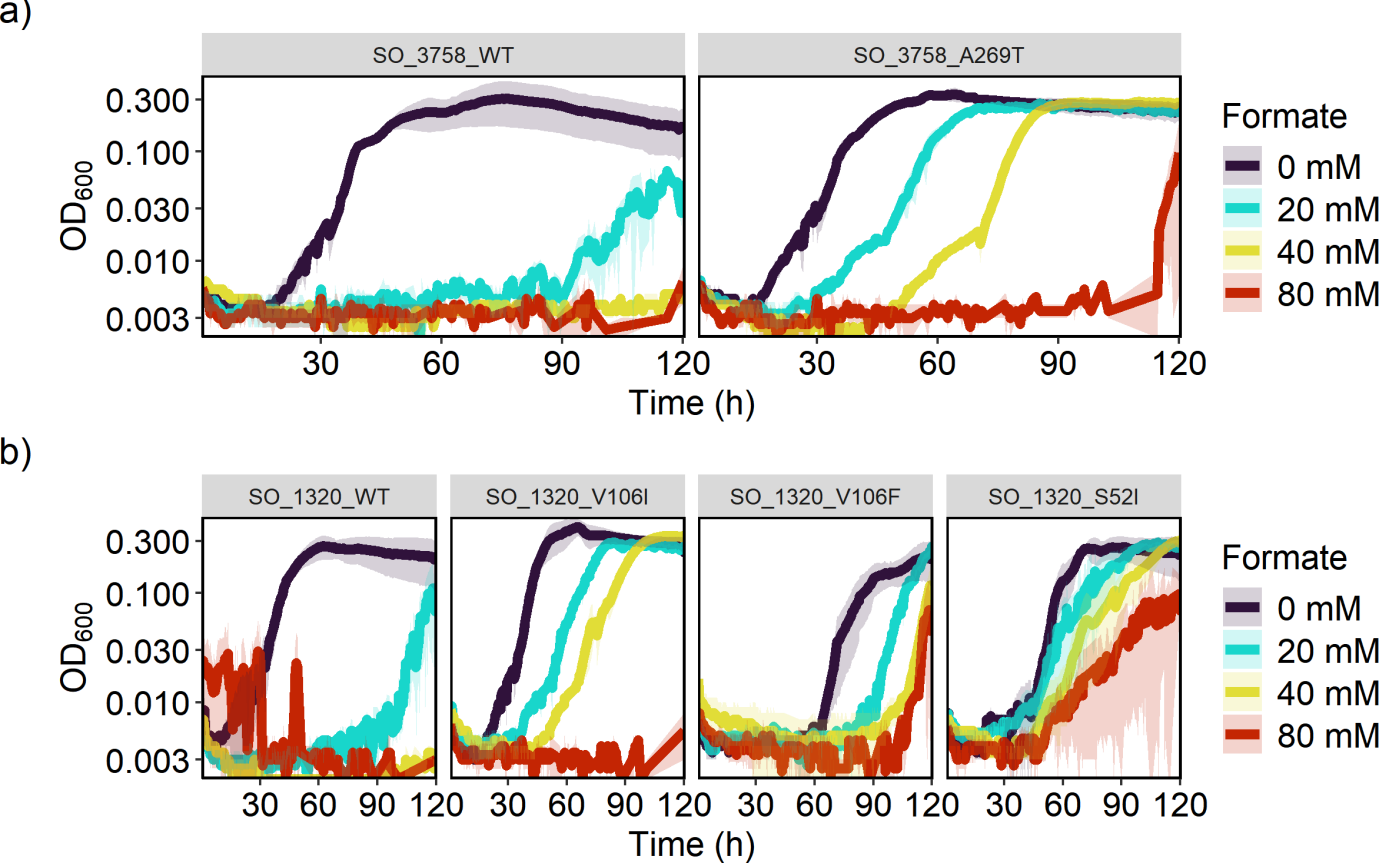
Growth curves of WT *S. oneidensis* harboring an IPTG-inducible expression vector containing WT or mutant versions of SO_3758 (**a**) or SO_1320 (**b**) in 200 µL minimal lactate medium with 100 µM IPTG and varying formate concentrations. Each condition was tested in triplicate, and standard deviation is displayed as transparent ribbons.

To confirm that amino acid changes in SO_3758 and SO_1320 were responsible for enhanced formate tolerance, we attempted to confer formate tolerance to WT *S. oneidensis* by overexpressing the wild-type or mutant sequences. Wild-type and mutant genes were cloned from the wild-type and formate-tolerant strains, respectively, and placed on vector pRL814 under control of an IPTG-inducible promoter. The vectors carrying the genes for overexpression were transformed into *E. coli* and subsequently transferred to WT *S. oneidensis* via conjugation. The *S. oneidensis* strains harboring SO_3758, SO_3758_A269T, SO_1320, SO_1320_V106I, SO_1320_V106F, or SO_1320_S52I were cultivated in 200 µL of minimal lactate medium with varying formate concentrations, and the WT or mutant gene was overexpressed with 100 µM IPTG (Fig. 5). All strains, whether expressing WT or mutant genes, were capable of growth without formate. The strains overexpressing the WT versions of SO_3758 and SO_1320 were only able to grow robustly in the no formate conditions, similar to the WT with no overexpression. The strains expressing any of the mutant sequences grew in elevated formate levels up to 40 mM with a final OD_600,_ similar to growth in the medium without formate. None of the strains tolerated 80 mM formate, indicating that other mutations accumulated during the evolution of the formate-tolerant strains could enhance their ability to withstand elevated levels of formate. This analysis confirmed that the identified mutations in SO_3758 and SO_1320 improved formate tolerance of WT *S. oneidensis*.

We performed multiple sequence alignments of SO_3758 and SO_1320 from multiple strains of *Shewanella* to determine how common the observed residue changes are across the genus. The putative sodium-dependent bicarbonate transporter encodes an alanine at the residue equivalent to residue 269 in *S. oneidensis* in 39 of the 40 strains examined (Fig. S3; Table S4). One strain, *Shewanella sp*. SNU WT4, encodes glycine in this position. Substitution of alanine for threonine is not observed in any of the *Shewanella* species examined. The multiple sequence alignment of SO_1320-like proteins from other *Shewanella* species (Table S5) reveals that residue 52 is often a serine, lysine, or arginine, but not an isoleucine, as observed in one of our mutant strains (Fig. S4a). The substitution of a polar or charged residue to a nonpolar, uncharged residue may play a role in the mechanism of improved formate tolerance for the SO_1320_V106I mutant. Residue 106 is always a valine in the *Shewanella* species examined (Fig. S4b), indicating that the substitution of this residue for something bulkier may contribute to the unknown formate tolerance mechanism of some SO_1320 mutants.

To predict the effect of the A269T substitution in the putative transporter encoded by SO_3758, we modeled the binding of bicarbonate and formate to the WT and mutant versions of the protein. To predict the binding site for modeling substrate binding, the *S. oneidensis* SO_3758 amino acid sequence was aligned with the only Sbt-like sodium-dependent bicarbonate transporter core domain with a solved structure (SbtA from cyanobacteria). The alignment revealed that A269 in SO_3758 corresponds to residue S323 in SbtA (Fig. S5). While S323 is not directly involved in binding sodium or bicarbonate in SbtA, it is the single residue that separates the two binding pockets, with S322 binding sodium and S324 binding bicarbonate (50, 51). These substrate-binding serines are conserved in SO_3758, with SO_3758 S268 corresponding to SbtA S322 and SO_3758 S270 corresponding to SbtA S324 (Fig. S5). The alignment revealed that SO_3758 residues V99, S100, S270, and I272 could be part of a bicarbonate binding pocket.

**Fig 6:**
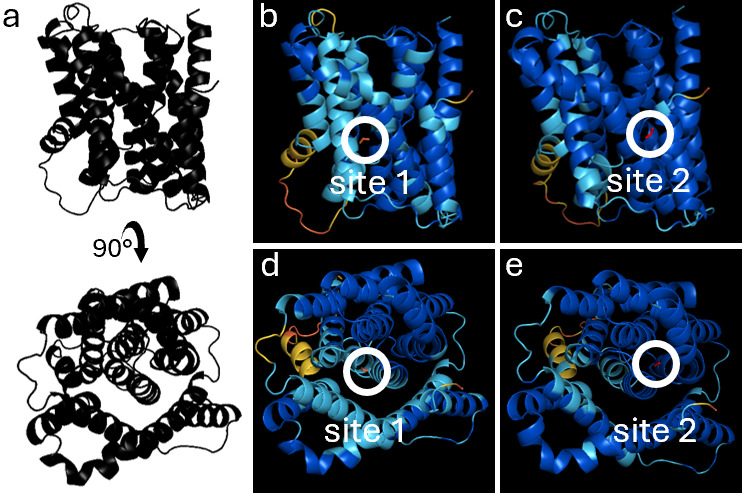
Representations of pLDDT scores from SO_3758 AlphaFold structural predictions. Residue color depicts pLDDT score. Dark blue represents a very high score (pLDDT >90). Light blue represents a confident score (90 > pLDDT > 70). Yellow represents a low score (70 > pLDDT > 50). Orange represents a very low score (pLDDT <50). (**a**) General structure of SO_3758 shown at two angles. View 1 is shown in panels b and c. View 2 is shown in panels d and e. (**b**) View 1 of WT SO_3758. Formate is colored red and shown binding to site 1. (**c**) View 1 of SO_3758_A269T. Formate is colored red and shown binding to site 2. (**d**) View 2 of WT SO_3758. Formate is colored red and shown binding to site 1. (**e**) View 2 of SO_3758_A269T. Formate is colored red and shown binding to site 2.

**Fig 7:**
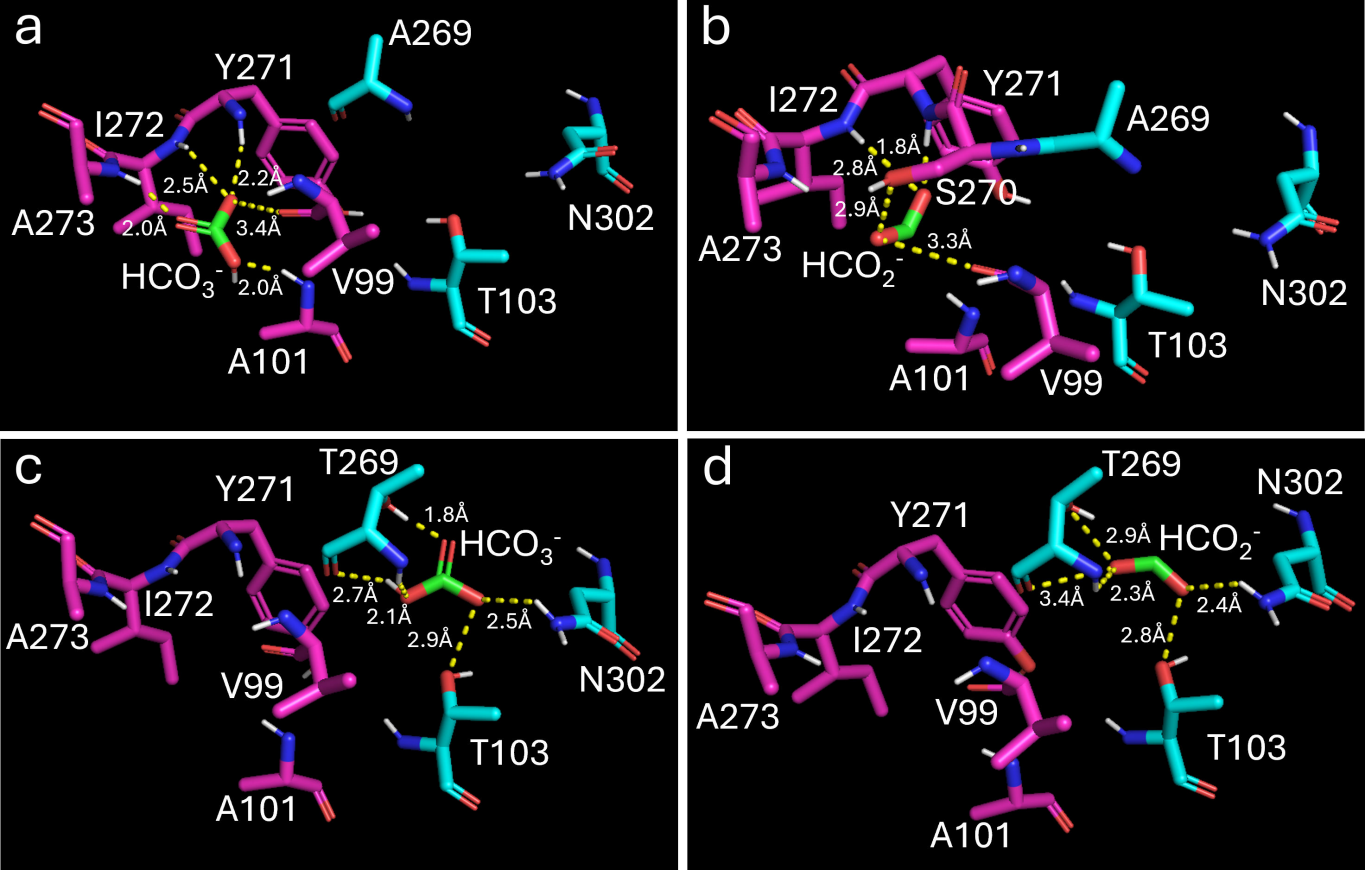
Modeled binding of bicarbonate (HCO_3_^-^) to (a) SO_3758_WT and (c) SO_3758_A269T and of formate (HCO_2_^-^) to (b) SO_3758_WT and (d) SO_3758_A269T. Residues in the native bicarbonate binding site (site 1) are shown in pink, and residues in site 2 are shown in cyan. Formate and bicarbonate are shown in green. Nitrogen atoms are colored blue, oxygen atoms are colored red, and hydrogen atoms are colored white. Polar interactions between bicarbonate or formate and interacting residues are shown as yellow dotted lines and are labeled with the distance between the interacting atoms. Both bicarbonate and formate form polar interactions with the mutated residue (T269) in SO_3758_A269T, which contribute to the tighter binding of these substrates in the mutated protein.

Structural models of SO_3758_WT and SO_3758_A269T were generated using AlphaFold (44), and the binding of bicarbonate and formate was predicted with Autodock Vina (45). The predicted local distance difference test (pLDDT) scores were calculated for each residue in the AlphaFold model and are depicted in Fig. 6, indicating the level of confidence in the structural prediction. The putative binding pocket residues had pLDDT scores of high (90 > pLDDT > 70) to very high (pLDDT >90) confidence. The docking grid was set to encompass residues predicted by sequence alignment to be part of a binding pocket (V99, S100, S270, and I272), A269 or T269, and nearby residues. The modeling of bicarbonate binding to SO_3758_WT predicts that it forms polar interactions with V99, A101, Y271, I272, and A273 in the predicted binding site, referred here as site 1 (Fig. 7a). Bicarbonate binding to site 1 has a predicted binding affinity of −3.2 kcal mol^−1^ (Table 1). Formate is also predicted to bind to site 1 in SO_3758_WT, forming polar interactions with V99, S270, Y271, and I272 and having a weaker predicted binding affinity of −2.3 kcal mol^−1^ (Fig. 7b).

**TABLE 1:** Predicted binding affinities of bicarbonate and formate to site 1 and site 2 of WT and variant versions of SO_3758

	Binding affinity to SO_3758_WT		Binding affinity to SO_3758_A269T	
	Site 1 (kcal mol^−1^)	Site 2 (kcal mol^−1^)	Site 1 (kcal mol^−1^)	Site 2 (kcal mol^−1^)
Bicarbonate	−3.2	−2.9	−3.4	−3.7
Formate	−2.3	NA	−2.9	−3.1

When modeled with SO_3758_A269T, bicarbonate is still predicted to bind to site 1 and form polar interactions with the same residues with a slightly stronger binding affinity of −3.4 kcal mol^−1^. However, the greatest predicted bicarbonate binding affinity of −3.7 kcal mol^−1^ is found in a binding pocket with the mutated T269 residue, as well as residues T103 and N302, here referred to as site 2 (Fig. 7c). This indicates that the A269T mutation could alter the predicted bicarbonate binding site 1 slightly to promote tighter binding of bicarbonate, even though residue 269 is not itself a part of site 1. More interestingly, it shows that the change in residue 269 alters site 2 to increase the affinity for bicarbonate. Finally, when formate binding is modeled with SO_3758_A269T, the greatest predicted binding affinity (−3.1 kcal mol^−1^) is also in site 2 with T103, N302, and the changed residue T269 (Fig. 7d). This brings the predicted binding affinity of formate much closer to that of bicarbonate in site 1 of the unmodified SO_3758 protein (−3.2 kcal mol^−1^), suggesting that formate is a suitable substrate for the variant. The predicted interaction of formate with threonine 269 in SO_3758_A269T but not with alanine in SO_3758_WT also suggests that the mutation resulting in this amino acid change could be beneficial in promoting formate binding.

The two binding sites are predicted to bind both substrates in SO_3758_A269T, but formate is not predicted to bind to site 2 in SO_3758_WT (Table 1). Site 1 binds both bicarbonate and formate more strongly than site 2. The opposite is true for SO_3758_A269T, where site 2 with residue threonine 269 binds both substrates more strongly and increases the predicted binding affinity of formate to a similar level that is predicted for bicarbonate binding in the wild-type protein. Based on this modeling, we considered that a plausible explanation for our results is that the A269T change in SO_3758 provided greater formate efflux capacity to prevent the toxic accumulation of formate inside the cells. The modeling results were not consistent with the hypothesis that the A269T change in SO_3758 reduced formate affinity to decrease formate import.

**Fig 8:**
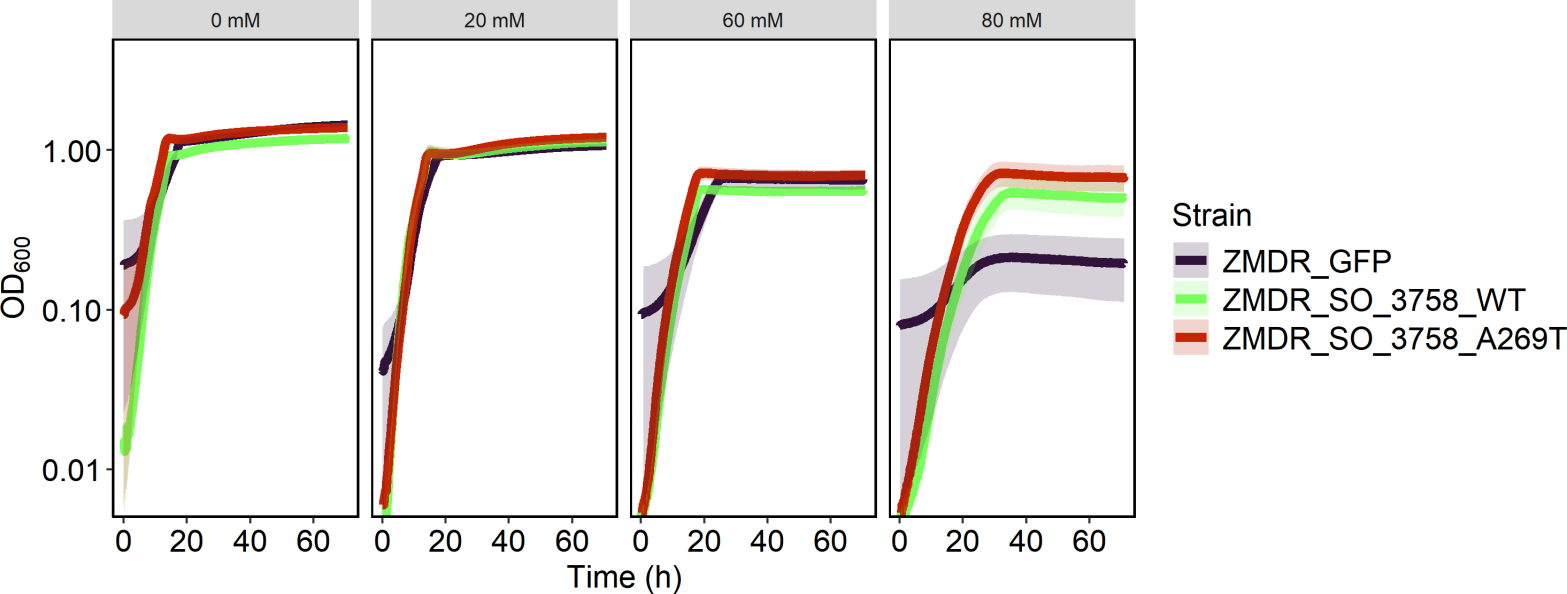
Growth curves of *Z. mobilis* ZMDR harboring an IPTG-inducible expression vector containing WT or mutant versions of SO_3758 or the empty vector in 200 µL ZRMG medium with varying formate concentrations. Each condition was tested in triplicate, and standard deviations are depicted as transparent ribbons.

To determine whether SO_3758_A269T could also confer formate tolerance to other species, we expressed SO_3758_WT and SO_3758_A269T in *Zymomonas mobilis*. This organism natively has greater formate tolerance than *S. oneidensis* and is an ethanol producer, making it a promising bio-production platform (52, 53). To determine whether SO_3758_A269T could improve formate tolerance in *Z. mobilis*, the organism was grown in rich medium with various formate concentrations either with the empty vector (pRL814), pRL814-SO_3758, or pRL814-SO_3758_A269T (Fig. 8). With its greater native formate tolerance, *Z. mobilis* carrying the empty vector was able to maintain similar growth in 20 mM and 60 mM formate as without formate. However, growth of the empty vector strain was impacted at 80 mM formate. The basal expression of either the WT or mutant versions of SO_3758 allowed *Z. mobilis* to maintain similar growth across all formate concentrations, indicating that both SO_3758_WT and SO_3758_A269T have the ability to improve formate tolerance in *Z. mobilis*. Inducing expression of either the WT or mutant versions of SO_3758 was toxic for *Z. mobilis* (data not shown).

The effects of mutations in DUF2721-containing protein SO_1320 are more difficult to ascertain. While the functions of both DUF2721 and SO_1320 are unknown, some clues exist to ascertain the protein’s potential role. Both YadS and SO_1328, expressed from the same putative operon as SO_1320, are part of membrane protein families. YadS is annotated as a trimeric intracellular cation (TRIC) channel, and SO_1318 is annotated as a nuclear transport factor 2-like (NTF2) protein (54). The proximity of SO_1320 to these membrane-bound transporter proteins could be an indicator that it shares a similar function.

In an attempt to elucidate the potential function of SO_1320, the amino acid sequence was analyzed using DeepGO, a deep learning tool which predicts the localization and function of a protein based on its sequence (42). DeepGO predicted that SO_1320 is localized to the cell membrane but was unable to predict its function, likely due to a lack of experimentally characterized homologs (Table S6). Further analysis of the SO_1320 amino acid sequence by DeepTMHMM (43), a tool for predicting transmembrane proteins using deep neural networks, reiterates the protein’s probable localization to the cell membrane, finding three transmembrane helices (Fig. S6). These results suggest that SO_1320 is associated with the cellular membrane, although it is still unclear what function it serves. Potential formate tolerance mechanisms of SO_1320 variants could include enhanced active formate efflux, decreased active formate influx, or changes in membrane properties altering passive formate diffusion.

To determine if SO_1320 could bind formate, the wild-type and variant versions of SO_1320 were modeled using AlphaFold (44), and formate binding was predicted with AutoDock Vina (45). SO_1320 did not appear to have convincing formate binding activity as the best predicted binding affinity was −2.1 kcal mol^−1^ at a site on the protein surface (Fig. S7). Modeling of formate binding to SO_1320_V106I, SO_1320_V106F, and SO_1320_S52I calculated the predicted binding affinities to formate of −2.0 kcal mol^−1^, -2.1 kcal mol^−1^, and −2.1 kcal mol^−1^, respectively. The weak predicted binding affinity, the location of the predicted binding, and the lack of improvement to the predicted binding affinity in the variants suggest that neither SO_1320 nor its variants act as formate transporters; however, it does not eliminate the possibility that it could be a formate channel or that it could form a multimeric complex with a formate transporter, whose substrate binding does not involve SO_1320 directly.

## DISCUSSION

This work describes the evolution of seven formate-tolerant *S. oneidensis* strains for use in systems subjected to high formate concentrations. Two notable mutations arose during the evolution of *S. oneidensis* for greater formate tolerance. The first is a mutation in a putative sodium-dependent bicarbonate transporter SO_3758, resulting in an amino acid change of alanine 269 to a threonine. Every strain exhibiting this mutation in SO_3758 (MGC002, MGC005, and MGC006) shared the same A269T substitution and was able to withstand greater formate concentrations than the parent strains. The second gene with mutations attributed to greater formate tolerance was SO_1320 encoding a protein of unknown function. Amino acid changes occurred in residue 106, changing the native valine to either isoleucine (MGC007) or phenylalanine (MGC003 and MGC004), and in residue 52 (MGC008), changing the native serine to isoleucine. The evolved strains gained the capacity to grow robustly on 100 mM formate in minimal lactate medium, while the parent strains they are derived from were only capable of growth on 10 mM formate.

Strains with the variant SO_1320_V106I had a shorter lag phase than strains with the variant SO_3758_A269T, although all strains grew to a similar final OD_600_ (Fig. 5). Therefore, the onset of formate tolerance benefits from the SO_1320 mutations occurred earlier than those from the SO_3758 mutation. The extra delay in growth of strains with the A269T substitution in SO_3758 may be related to the mechanism by which it provides formate tolerance. Modeling of formate binding to the WT and variant versions of the protein encoded by SO_3758 revealed that the conversion of alanine 269 to threonine strengthened the predicted binding affinity of formate to a site deeper in the binding pocket (site 2). The predicted binding affinity of both bicarbonate and formate in site 2 of SO_3758_A269T is stronger than the binding of both substrates to both site 1 and site 2 in the wild-type protein. The A269T change could allow formate to bind to the altered protein with a similar affinity as bicarbonate binding to the wild-type protein. If the protein encoded by SO_3758 is a bicarbonate transporter, as suggested by the annotation, our results suggest that formate could be a suitable alternative substrate for the A269 variant. The directionality of SO_3758 bicarbonate transport is not known; however, the modeling results are more consistent with the hypothesis that the protein is an efflux pump. If SO_3758 was a bicarbonate importer, improved formate binding would lead to a decrease in formate tolerance resulting from faster accumulation of toxic formate concentrations intracellularly.

Further evidence that SO_3758 is directly involved in formate tolerance is observed during expression in *Z. mobilis*. Interestingly, SO_3758_WT confers similar formate tolerance as SO_3758_A269T in this organism, an observation that differs from their performance in *S. oneidensis. Z. mobilis* does not have a version of the Sbt-like sodium-dependent bicarbonate transporter (55). This may suggest that the introduction of even the WT SO_3758, which modeling results suggest binds formate more weakly than SO_3758_A269T, can provide an effective mechanism for formate tolerance, which is otherwise absent. The ability to transfer improvement of formate tolerance to other species through expression of a single gene may be useful in engineering a variety of bacterial biosynthetic hosts that are limited by formate toxicity. Additionally, further protein engineering could improve on the single A269T substitution and further improve formate tolerance.

The roles of mutations in DUF2721-containing protein SO_1320 are less clear because neither this domain nor this protein have any known characterized homologs. SO_1320 is in a putative operon with genes encoding a membrane protein channel or membrane transport protein (*yadS* and SO_1318), and sequence analysis tools predict three transmembrane helices. These clues suggest that SO_1320 is also a membrane protein. Modeling of formate binding to SO_1320 and its variants indicates that the protein does not bind formate as a transporter might. While it is still unclear how SO_1320 variants are providing cells with greater formate tolerance, our analysis does not rule out the possibilities that SO_1320 is involved in membrane stabilization or acts as a formate channel. Additionally, SO_1320 could be one subunit in a multimeric formate transporter that binds formate in a different subunit. It is also possible that formate binding by SO_1320 could not be captured by our modeling techniques.

Expression of mutant genes identified in this study in the *S. oneidensis* WT background on minimal lactate medium increased formate tolerance twofold. The ability to reconstruct the formate-tolerant phenotype through expression of these altered proteins further confirms their role in the evolved strains. The simple point mutations that lead to these growth improvements provide two easily accessible methods for transferring formate tolerance to *S. oneidensis* and other organisms. This could be a valuable tool in engineering biocatalysts that would benefit from reduced intracellular formate or bicarbonate levels, such as in organisms in which these compounds block metabolic activity prematurely due to toxicity.

## Data Availability

All raw data, code, and models used in this study are available upon request. Sequencing data associated with this project are available in the NCBI Sequence Read Archive (SRA) under accession number PRJNA1228652.

## SUPPLEMENTAL MATERIAL

Supplemental material aem.01968-24-s0001.docx Figures S1 to S7; Tables S1 to S6.

ASM does not own the copyrights to Supplemental Material that may be linked to, or accessed through, an article. The authors have granted ASM a non-exclusive, world-wide license to publish the Supplemental Material files. Please contact the corresponding author directly for reuse.
