# Supplementary material for: A small number of point mutations confer formate tolerance in Shewanella oneidensis
Source: Appl Environ Microbiol. 2025 Apr 10;91(5):e01968-24. doi: 10.1128/aem.01968-24 (PMC12093963; doi:10.1128/aem.01968-24)
Supplement: Supplemental material — Figures S1 to S7; Tables S1 to S6. [file aem.01968-24-s0001.docx]

**Supplemental Information**

A small number of point mutations confer formate tolerance in *Shewanella oneidensis*

Megan C Gruenberg Cross^a^, Elhussiny Aboulnaga^a^, and Michaela A TerAvest^a^

^a^Department of Biochemistry and Molecular Biology, Michigan State University, East Lansing, MI, USA


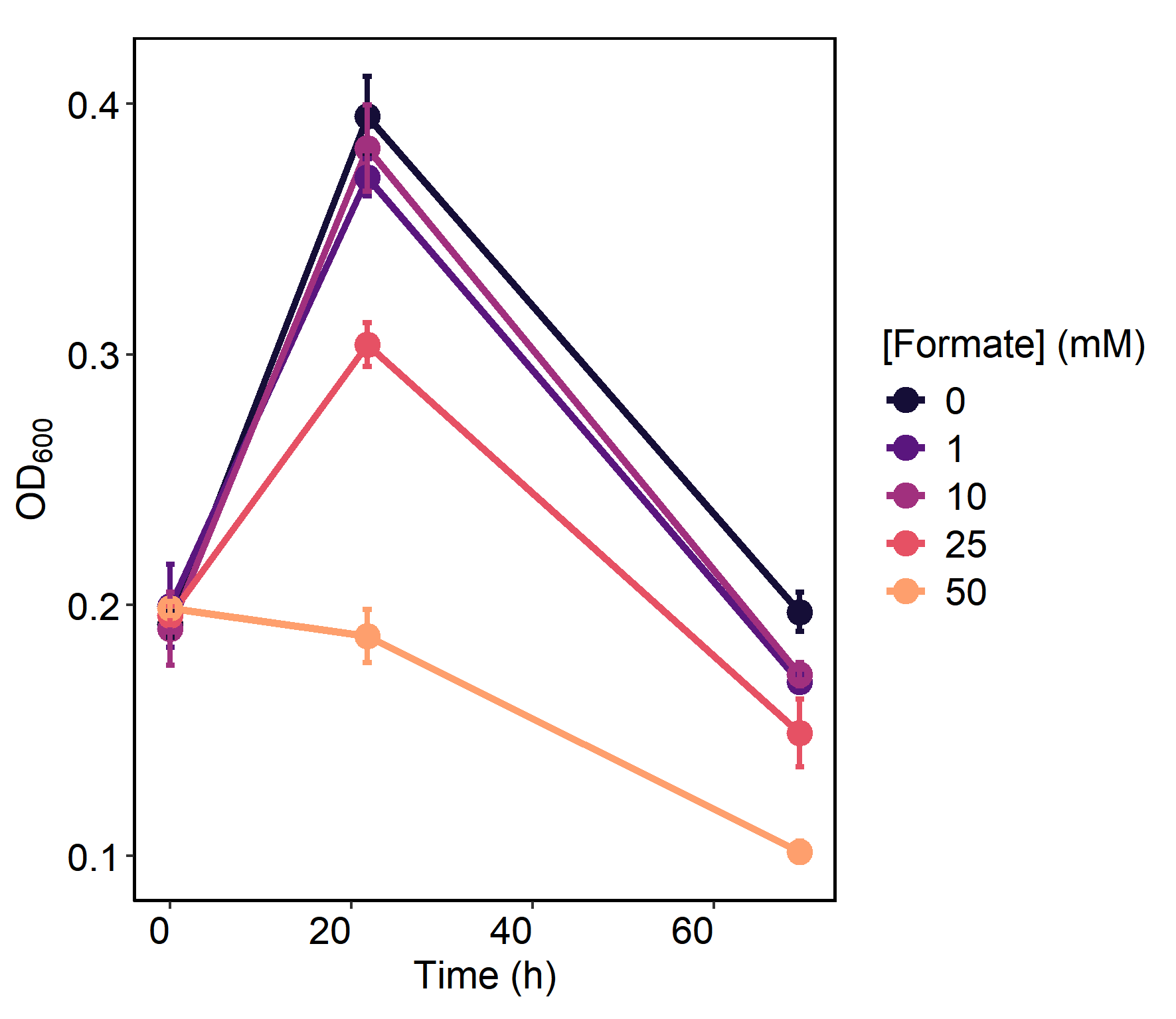


**Figure S1.** Anaerobic growth curves of MR-1 in 80 ml high HEPES minimal lactate media, 50 mM fumarate, and varying concentrations of formate. Culturing took place in serum bottles sparged with N_2_ for ten minutes. All conditions were tested in triplicate and standard deviation from replicates are displayed with error bars.


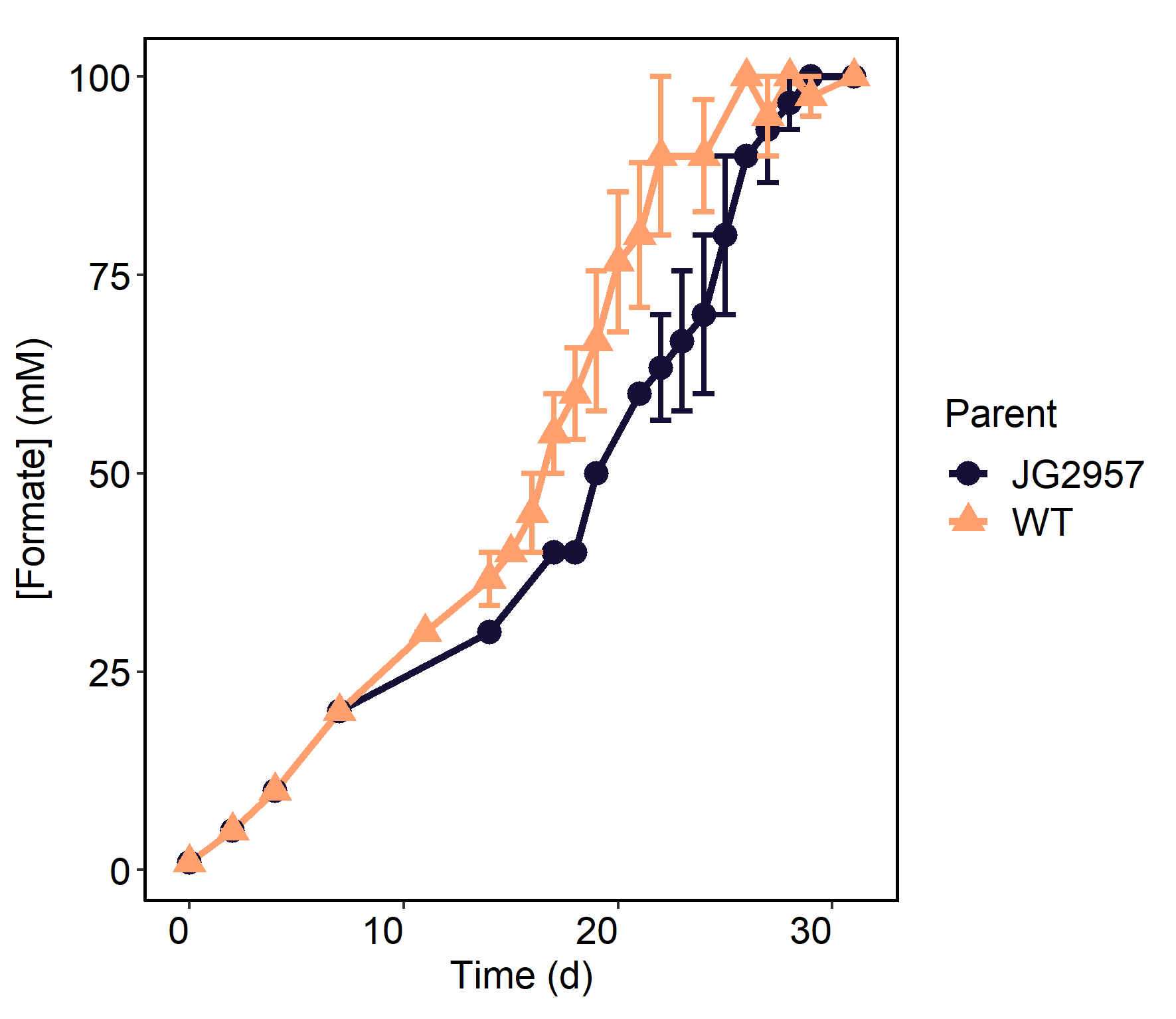


**Figure S2.** Time that each strain (four replicates for WT and three replicates for JG2957) required for subculturing with increasing concentrations of formate. Error bars indicate the standard deviation.

**
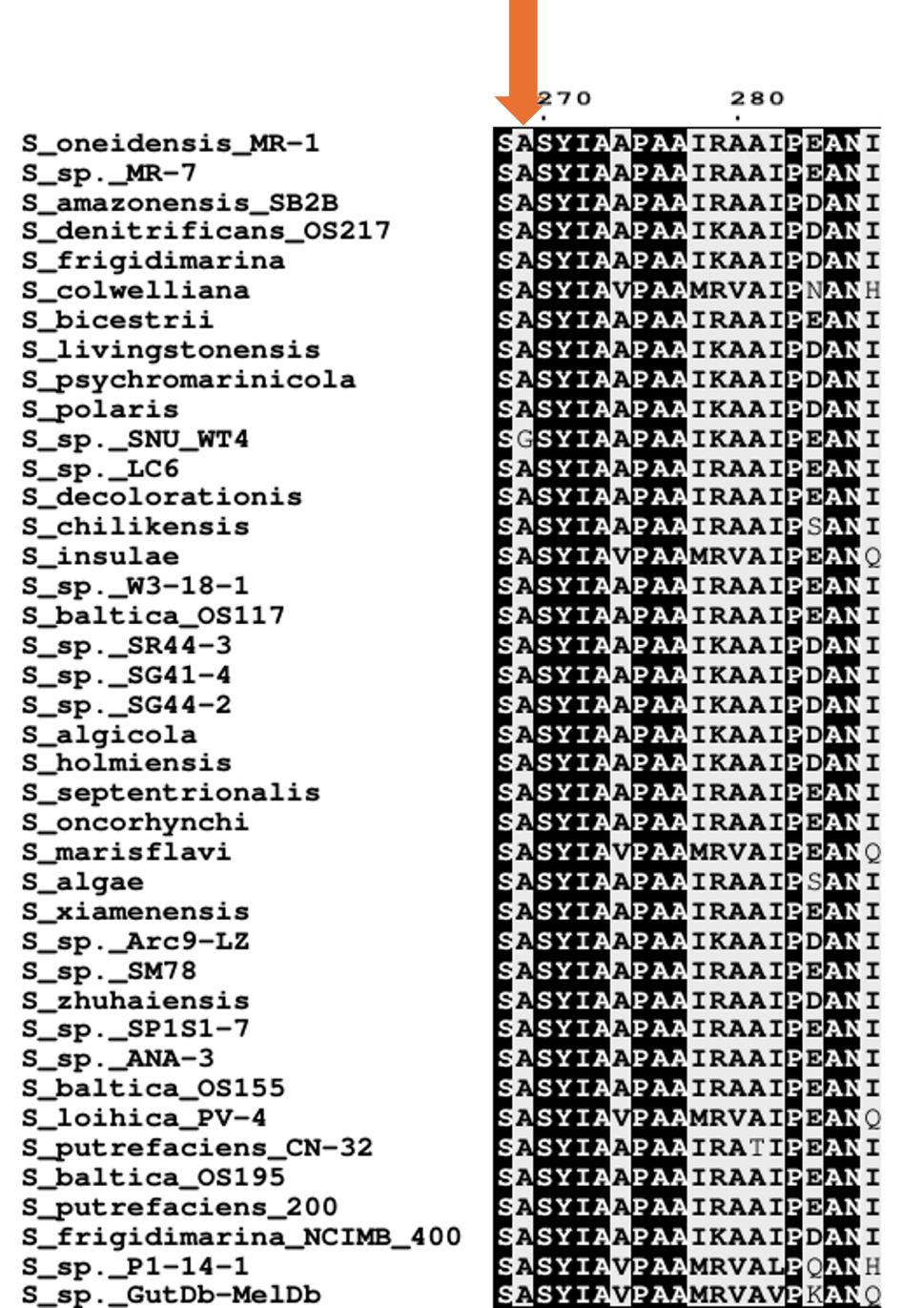
**

**Figure S3.** Multiple sequence alignment of sodium-dependent bicarbonate transporters from 40 *Shewanella* species. Residue numbers correspond to the residues from SO_3758 in *S. oneidensis* MR-1. Arrow is pointing to residue 269. All residues in this position are an alanine, except for in *Shewanella sp*. SNU WT4 in which the corresponding residue is a glycine.


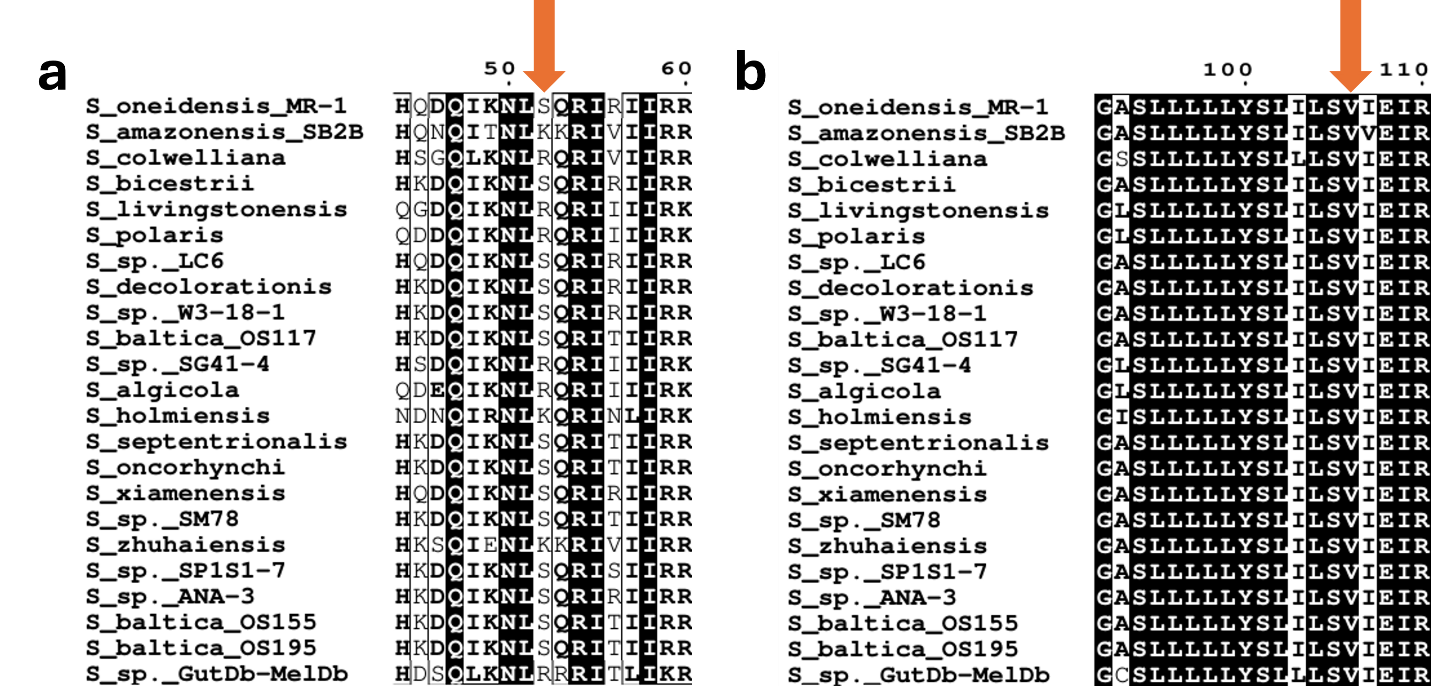


**Figure S4.** Multiple sequence alignment of SO_1320-like proteins from 23 *Shewanella* species. Residue numbers correspond to the residues from SO_1320 in *S. oneidensis* MR-1. a) Arrow is pointing to residue 52. All residues in this position are a serine, lysine, or arginine. b) Arros is pointing to residue 106. All residues in this position are a valine.

**
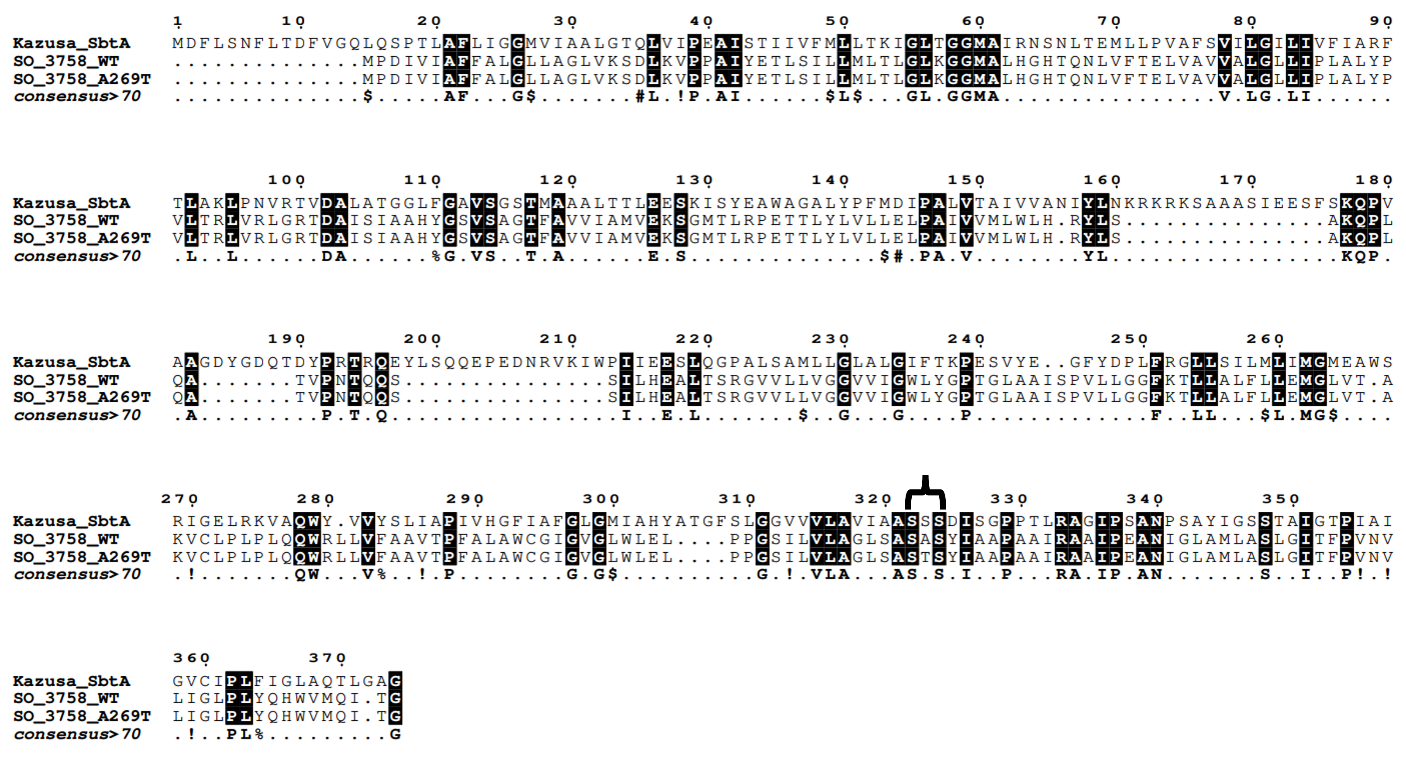
**

**Figure S5.** Multiple sequence alignment of cyanobacteria SbtA, WT SO_3758, and SO_3758_A269T. Bracket highlights residues A269 from WT SO_3758, T269 from SO_3758_A269T, and corresponding cyanobacteria SbtA residue S323, as well as conserved flanking serine residues involved in the binding of bicarbonate and sodium.


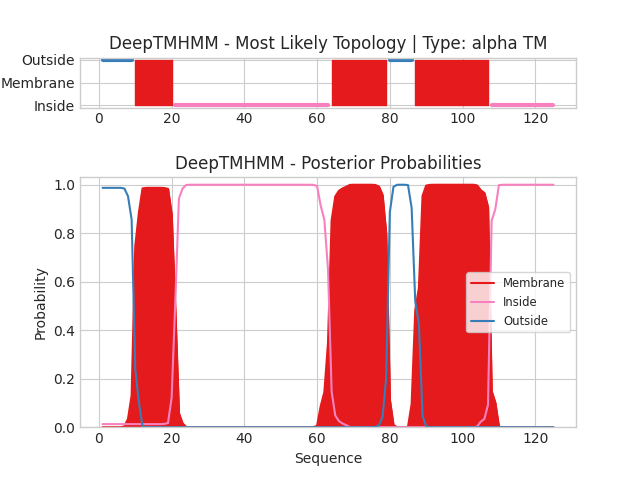


**Figure S6.** SO_1320 structure prediction by DeepTMHMM.


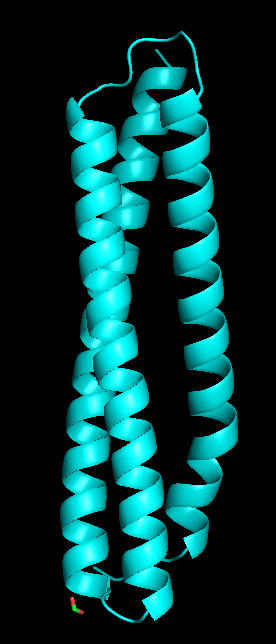


**Figure S7.** Predicted binding of formate to SO_1320 by AutoDock Vina.

**Table S1.** Strains and plasmids used in this study.

| Strain | Description | Reference |
| --- | --- | --- |
| *Parent strains* |  |  |
| *S. oneidensis* MR-1 | Wild-type *S. oneidensis* | Lab stock |
| JG2957 | *S. oneidensis* with deletion of SO_0101-SO_0103, SO_4509-SO_4511, and SO_4513-SO_4515 | (1) |
| JG2955 | *S. oneidensis* with deletion of SO_4509-SO_4511 and SO_4513-SO_4515 | (1) |
| *E. coli* WM3064 | Conjugal transfer donor, DAP auxotroph | Lab stock |
| *E. coli* WM6026 | Conjugal transfer donor, DAP auxotroph | Lab stock |
| *Z. mobilis* ZMDR | *Z. mobilis* ATCC 31281 Δcas3 (zmo0681) Δmrr (zmo0028) ΔhsdSc (zmo1933) ΔhsdSp (zmop32x028) | (2) |
| *Formate-tolerant strains* | |  |
| MCG001 | Formate-tolerant strain evolved from *S. oneidensis* MR-1. (Line A Colony 3) | This study |
| MGC002 | Formate-tolerant strain evolved from *S. oneidensis* MR-1. (Line B Colony 1) | This study |
| MGC003 | Formate-tolerant strain evolved from *S. oneidensis* MR-1. (Line C Colony 3) | This study |
| MGC004 | Formate-tolerant strain evolved from *S. oneidensis* MR-1. (Line D Colony 1) | This study |
| MGC005 | Formate-tolerant strain evolved from *S. oneidensis* JG2957. (Line A Colony 2) | This study |
| MGC006 | Formate-tolerant strain evolved from *S. oneidensis* JG2957. (Line B Colony 1) | This study |
| MGC007 | Formate-tolerant strain evolved from *S. oneidensis* JG2957. (Line C Colony 2) | This study |
| MGC008 | Formate-tolerant strain evolved from *S. oneidensis* JG2955. (Colony 2) | This study |
| Plasmids |  |  |
| pRL814 | Broad host plasmid expressing GFP under T7A1-034 promoter, lacI, spectinomycin resistance | (3) |
| pRL814-SO_3758 | WT SO_3758 expression vector, derivative of pRL814, spectinomycin resistance | This study |
| pRL814-SO_3758_A269T | SO_3758_A269T expression vector, derivative of pRL814, spectinomycin resistance | This study |
| pRL814-SO_1320 | WT SO_1320 expression vector, derivative of pRL814, spectinomycin resistance | This study |
| pRL814-SO_1320_V106I | SO_1320_V106I expression vector, derivative of pRL814, spectinomycin resistance | This study |
| pRL814-SO_1320_V106F | SO_1320_V106F expression vector, derivative of pRL814, spectinomycin resistance | This study |
| pRL814-SO_1320_S52I | SO_1320_S52I expression vector, derivative of pRL814, spectinomycin resistance | This study |

**Table S2.** Primers used in this study.

| Primer | Sequence (5’->3’) | Description |
| --- | --- | --- |
| MG_203 | ctttaagaaggagatatacatatgccagatattgtcattgca | SO_3758 cloning fwd |
| MG_204 | aggaattcgatatcattatttatcatcatcatctttgtaatctcccgtaatttgcattac | SO_3758 cloning rev |
| MG_205 | ctttaagaaggagatatacatatgcatgtttcattaacaac | SO_1320 fwd |
| MG_206 | aggaattcgatatcattatttatcatcatcatctttgtaatctttactcatctctttgat | SO_1320 rev |

**Table S3.** Mutations in formate-tolerant strains identified by Breseq. Mutations in SO_3758 are highlighted in green. Mutations in SO_1320 are highlighted in purple.

| strain | seq id | position | mutation | annotation | gene | description |
| --- | --- | --- | --- | --- | --- | --- |
| MGC001 | NC_004347 | 2,782,334 | T→C | intergenic (‑97/‑29) | *ppsA* ← / → *SO_RS12190* | phosphoenolpyruvate synthase/pyruvate, water dikinase regulatory protein |
|  | NC_004347 | 3,258,800 | Δ1 bp | intergenic (‑86/+14) | *SO_RS14530* ← / ← *SO_RS14535* | tRNA‑Glu/tRNA‑Glu |
|  | NC_004347 | 3,588,820 | N→T | ?94T (ACN→ACA) | *SO_RS16095* ← | IS256‑like element ISSod4 family transposase |
|  | NC_004347 | 3,928,289 | G→T | R213L (CGC→CTC) | *SO_RS17595* → | DUF599 domain‑containing protein |
|  | NC_004349 | 138,980 | Δ3 bp | coding (349‑351/1041 nt) | *SO_RS22825* ← | IS481‑like element ISSod13 family transposase |
| MCG002 | NC_004347 | 3,258,789 | N→T | intergenic (‑75/+25) | *SO_RS14530* ← / ← *SO_RS14535* | tRNA‑Glu/tRNA‑Glu |
|  | NC_004347 | 3,258,800 | Δ1 bp | intergenic (‑86/+14) | *SO_RS14530* ← / ← *SO_RS14535* | tRNA‑Glu/tRNA‑Glu |
|  | **NC_004347** | **3,906,124** | **G→A** | **A269T (GCC→ACC)** | ***SO_RS17510* →** | **sodium‑dependent bicarbonate transport family permease** |
| MGC003 | **NC_004347** | **1,374,567** | **C→A** | **V106F (GTC→TTC)** | ***SO_RS06120* ←** | **DUF2721 domain‑containing protein** |
|  | NC_004347 | 2,143,924 | (CCATGA)_10→11_ | coding (686/1185 nt) | *SO_RS09420* → | cation diffusion facilitator family transporter |
|  | NC_004347 | 3,258,800 | Δ1 bp | intergenic (‑86/+14) | *SO_RS14530* ← / ← *SO_RS14535* | tRNA‑Glu/tRNA‑Glu |
|  | NC_004347 | 3,355,020 | G→A | P74L (CCA→CTA) | *flhA* ← | flagellar biosynthesis protein FlhA |
|  | NC_004347 | 4,740,503 | G→A | W48* (TGG→TGA) | *SO_RS21100* → | alpha/beta hydrolase‑fold protein |
| MGC004 | **NC_004347** | **1,374,567** | **C→A** | **V106F (GTC→TTC)** | ***SO_RS06120* ←** | **DUF2721 domain‑containing protein** |
|  | NC_004347 | 2,143,924 | (CCATGA)_10→11_ | coding (686/1185 nt) | *SO_RS09420* → | cation diffusion facilitator family transporter |
|  | NC_004347 | 3,355,020 | G→A | P74L (CCA→CTA) | *flhA* ← | flagellar biosynthesis protein FlhA |
| MGC005 | NC_004347 | 2,353,953 | C→T | D334N (GAT→AAT) | *ptsP* ← | phosphoenolpyruvate‑‑protein phosphotransferase |
|  | NC_004347 | 3,258,800 | Δ1 bp | intergenic (‑86/+14) | *SO_RS14530* ← / ← *SO_RS14535* | tRNA‑Glu/tRNA‑Glu |
|  | **NC_004347** | **3,906,124** | **G→A** | **A269T (GCC→ACC)** | ***SO_RS17510* →** | **sodium‑dependent bicarbonate transport family permease** |
| MGC006 | NC_004347 | 2,871,939 | C→T | D23N (GAC→AAC) | *tolR* ← | protein TolR |
|  | **NC_004347** | **3,906,124** | **G→A** | **A269T (GCC→ACC)** | ***SO_RS17510* →** | **sodium‑dependent bicarbonate transport family permease** |
|  | NC_004349 | 138,980 | Δ3 bp | coding (349‑351/1041 nt) | *SO_RS22825* ← | IS481‑like element ISSod13 family transposase |
| MC007 | **NC_004347** | **1,374,567** | **C→T** | **V106I (GTC→ATC)** | ***SO_RS06120* ←** | **DUF2721 domain‑containing protein** |
|  | NC_004347 | 3,258,789 | N→T | intergenic (‑75/+25) | *SO_RS14530* ← / ← *SO_RS14535* | tRNA‑Glu/tRNA‑Glu |
|  | NC_004347 | 3,258,800 | Δ1 bp | intergenic (‑86/+14) | *SO_RS14530* ← / ← *SO_RS14535* | tRNA‑Glu/tRNA‑Glu |

**Table S4.** Accession numbers for SO_3758-like proteins from *Shewanella* species used for multiple sequence alignment.

| Strain | SO_3758-like Protein Accession Number |
| --- | --- |
| *Shewanella sp.* MR-7 | Q0HYE1 |
| *S. amazonensis* SB2B | WP_011758810.1 |
| *S. denitrificans* OS217 | WP_011495158.1 |
| *S. frigidimarina* | WP_059746641.1 |
| *S. colwelliana* | WP_028765319.1 |
| *S. bicestrii* | WP_089068392.1 |
| *S. livingstonensis* | WP_124732072.1 |
| *S. psychromarinicola* | WP_335686822.1 |
| *S. polaris* | A0A4Y5YIT4 |
| *S. sp.* SNU WT4 | WP_140933551.1 |
| *S. sp.* LC6 | WP_055647754.1 |
| *S. decolorationis* | WP_208661794.1 |
| *S. chilikensis* | WP_248952930.1 |
| *S. insulae* | WP_160795436.1 |
| *S. sp.* W3-18-1 | WP_011788237.1 |
| *S. baltica* OS117 | WP_011847664.1 |
| *S. sp.* SR44-3 | WP_182678237.1 |
| *S. sp.* SG41-4 | WP_182770046.1 |
| *S. sp.* SG44-2 | WP_011638698.1 |
| *S. algicola* | WP_188923402.1 |
| *S. holmiensis* | WP_261297115.1 |
| *S. septentrionalis* | WP_261272029.1 |
| *S. oncorhynchi* | WP_306684702.1 |
| *S. marisflavi* | A0AAC9XP07 |
| *S. algae* | WP_208146190.1 |
| *S. xiamenensis* | WP_172587612.1 |
| *S. sp.* Arc9-LZ | WP_101085283.1 |
| *S. sp.* SM78 | WP_263175828.1 |
| *S. zhuhaiensis* | WP_240589691.1 |
| *S. sp.* SP1S1-7 | WP_312000262.1 |
| *S. sp.* ANA-3 | WP_011715978.1 |
| *S. baltica* OS155 | WP_011847664.1 |
| *S. loihica* PV-4 | A3QCB9 |
| *S. putrefaciens* CN-32 | WP_011919907.1 |
| *S. baltica* OS195 | WP_006085954.1 |
| *S. putrefaciens* 200 | WP_278684720.1 |
| *S. frigidimarina* NCIMB 400 | WP_059746641.1 |
| *S. sp.* P1-14-1 | WP_055024770.1 |
| *S. sp.* GutDb-MelDb | A0A2I0DZQ2 |

**Table S5.** Accession numbers for SO_1320-like proteins from *Shewanella* species used for multiple sequence alignment.

| Strain | SO_1320-like Protein Accession Number |
| --- | --- |
| *S. amazonesis* SB2B | ABL99060.1 |
| *S. colwelliana* | WP_028762255.1 |
| *S. bicestrii* | WP_011623626.1 |
| *S. livingstonensis* | WP_124731811.1 |
| *S. polaris* | WP_137222385.1 |
| *S. sp.* LC6 | WP_037415657.1 |
| *S. decolorationis* | GLR33718.1 |
| *S. sp.* W3-18-1 | WP_011790301.1 |
| *S. baltica* OS117 | AEH13039.1 |
| *S. sp.* SG41-4 | WP_182773813.1 |
| *S. algicola* | WP_188925848.1 |
| *S. holmiensis* | WP_261297530.1 |
| *S. septentrionalis* | WP_006085052.1 |
| *S. oncorhynchi* | WP_012088590.1 |
| *S. xiamenensis* | BDQ67140.1 |
| *S. sp.* SM78 | WP_012088590.1 |
| *S. zhuhaiensis* | WP_126169388.1 |
| *S. sp.* SP1S1-7 | WP_006080752.1 |
| *S. sp.* ANA-3 | WP_011623626.1 |
| *S. baltica* OS155 | WP_006085052.1 |
| *S. baltica* OS195 | WP_006085052.1 |
| *S. sp.* GutDb-MelDb | WP_101090548.1 |

**Table S6.** DeepGO predictions for SO_1320.

| Gene Ontology | Description | Prediction Score |
| --- | --- | --- |
| Cellular Component |  |  |
| GO:0110165 | cellular anatomical entity | 0.392872 |
| GO:0016020 | membrane | 0.370649 |
| Molecular Function |  |  |
| Biological Process |  |  |

**References**

1. Kane AL, Brutinel ED, Joo H, Maysonet R, VanDrisse CM, Kotloski NJ, Gralnick JA. 2016. Formate Metabolism in *Shewanella oneidensis* Generates Proton Motive Force and Prevents Growth without an Electron Acceptor. J Bacteriol 198:1337–1346.

2. Lal PB, Wells F, Myers KS, Banerjee R, Guss AM, Kiley PJ. 2021. Improving Mobilization of Foreign DNA into *Zymomonas mobilis* Strain ZM4 by Removal of Multiple Restriction Systems. Appl Environ Microbiol 87.

3. Ghosh IN, Martien J, Hebert AS, Zhang Y, Coon JJ, Amador-Noguez D, Landick R. 2019. OptSSeq explores enzyme expression and function landscapes to maximize isobutanol production rate. Metab Eng 52:324–340.
